# Primary CD20‐positive mediastinal diffuse large B‐cell lymphoma

**DOI:** 10.1002/rcr2.668

**Published:** 2020-09-24

**Authors:** Wulyo Rajabto, Dimas Priantono

**Affiliations:** ^1^ Division of Hematology‐Medical Oncology, Department of Internal Medicine Dr. Cipto Mangunkusumo General Hospital/Faculty of Medicine Universitas Indonesia Jakarta Indonesia

**Keywords:** CD20‐positive, DLBCL, lymphoma, mediastinal

## Abstract

Primary mediastinal B‐cell lymphoma (PMBCL) is a rare tumour with different characteristics from other type of lymphomas. Clinical manifestations may vary and cause delay in diagnosis and management. We present a 22‐year‐old patient with symptoms of shortness of breath, weight loss, and night sweats. Laboratory studies only showed a markedly high lactate dehydrogenase (LDH) level and thoracic computed tomography (CT)scan revealed a large mediastinal mass. Core biopsy‐guided CT scan was performed and the pathological and immunohistochemistry established a PMBCL diagnosis. We administered Rituximab Dose‐Adjusted Etoposide Prednisolone Vincristine Cyclophosphamide Doxorubicin (R‐DA‐EPOCH) chemotherapy regimen and the patient responded well to treatment. This is an example of rare case of mediastinal lymphoma with challenges to overcome to achieve diagnostic and therapeutic success. Failure to differentiate PMBCL with other systemic diffuse large B‐cell lymphoma (DLBCL) could skew treatment algorithm and prevent optimal response. Administration of proper systemic therapy, especially in young, low‐risk patients could yield excellent outcome.

## Graphical abstract

Primary mediastinal B‐cell lymphoma (PMBCL) is a rare tumour with different characteristics from other type of lymphomas. Clinical manifestations may vary and cause delay in diagnosis and management. We reported a patient with PMBCL diagnosis and good response to treatment.

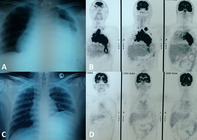

## Introduction

Mediastinal lymphoma accounts for more than half of mediastinal malignancies, making it the most common malignant tumour in this area [1]. However, most mediastinal lesions are usually an extension of an existing systemic lymphoma [2]. Primary mediastinal B‐cell lymphoma (PMBCL) only accounts for about 10% of diffuse large B‐cell lymphomas (DLBCL) [3] and 2–4% of all non‐Hodgkin lymphomas [4]. This type of tumour is commonly found in adolescence and young adulthood, with more incidence in females than males [3, 5]. Clinical manifestations vary widely, with dominant symptoms usually attributed to the effect of the tumour to adjacent organs.

## Case Report

**Figure 1:**
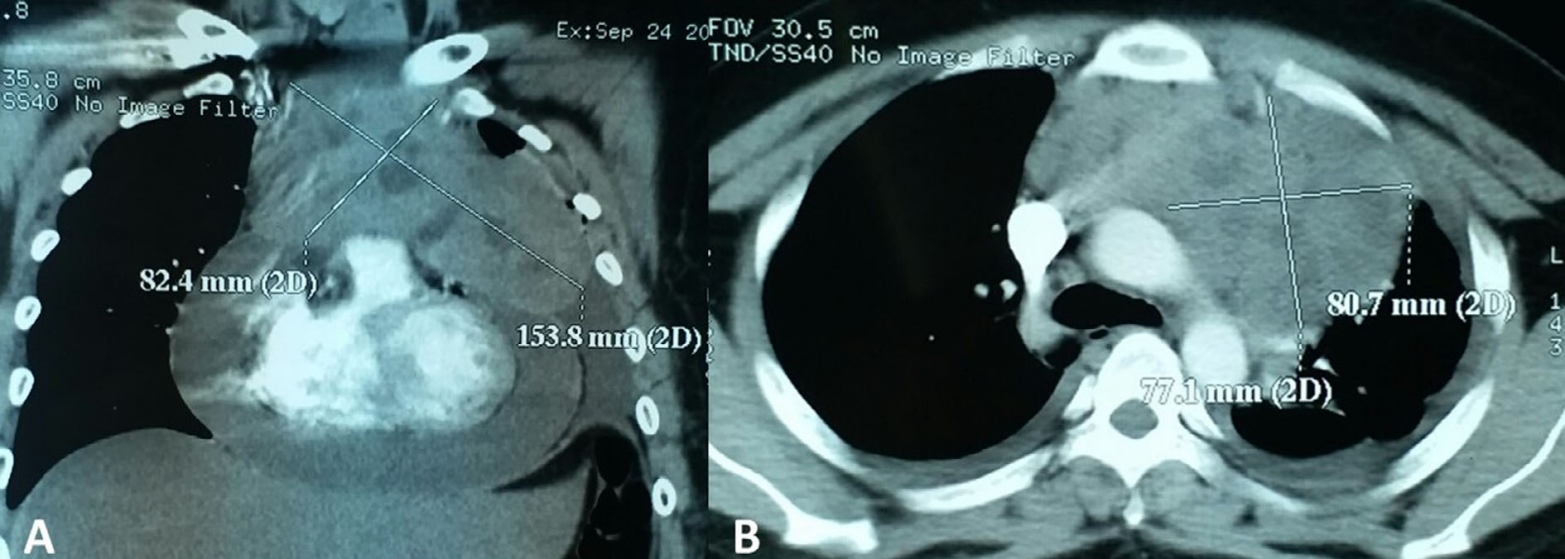
Mediastinal computed tomography (CT) scan showing the tumour. (A) Coronal image. (B) Axial image.

A 21‐year‐old male patient visited outpatient clinic Haematology‐Medical Oncology Department of Internal Medicine, Dr. Cipto Mangunkusumo General Hospital, with shortness of breath since three months before admission. He also experienced night sweats and weight loss of 5 kg. Laboratory studies only showed an elevated lactate dehydrogenase (LDH) level, 484 U/L (reference range: 125–220 U/L). Thoracic computed tomography (CT) scan revealed a large mediastinal mass (Fig. 1).

Whole body positron emission tomography (PET)–CT imaging was performed and showed a poorly demarcated hypermetabolic mass with a necrotic core located in the anterior mediastinum. The mass itself had displaced anterior mediastinal organs dorsally. The tumour also encased vascular structures, mainly brachiocephalic vein. Nodal involvement was found in multiple sites. Pericardial and bilateral pleural effusions were also present.

We performed a CT‐guided core biopsy procedure on the mass. Histopathological study found the tumour to be a non‐Hodgkin lymphoma. Further immunohistochemistry staining showed positive markers on CD20 (diffuse), CD30, CD79a, and Ki‐67 (50%). On the basis of imaging and histopathological findings, we established the diagnosis of stage II bulky CD20 (+) primary mediastinal DLBCL. According to age‐adjusted International Prognostic Index (aaIPI), this patient is categorized into low‐intermediate risk category, with estimated complete response rate of 78%.

**Figure 2:**
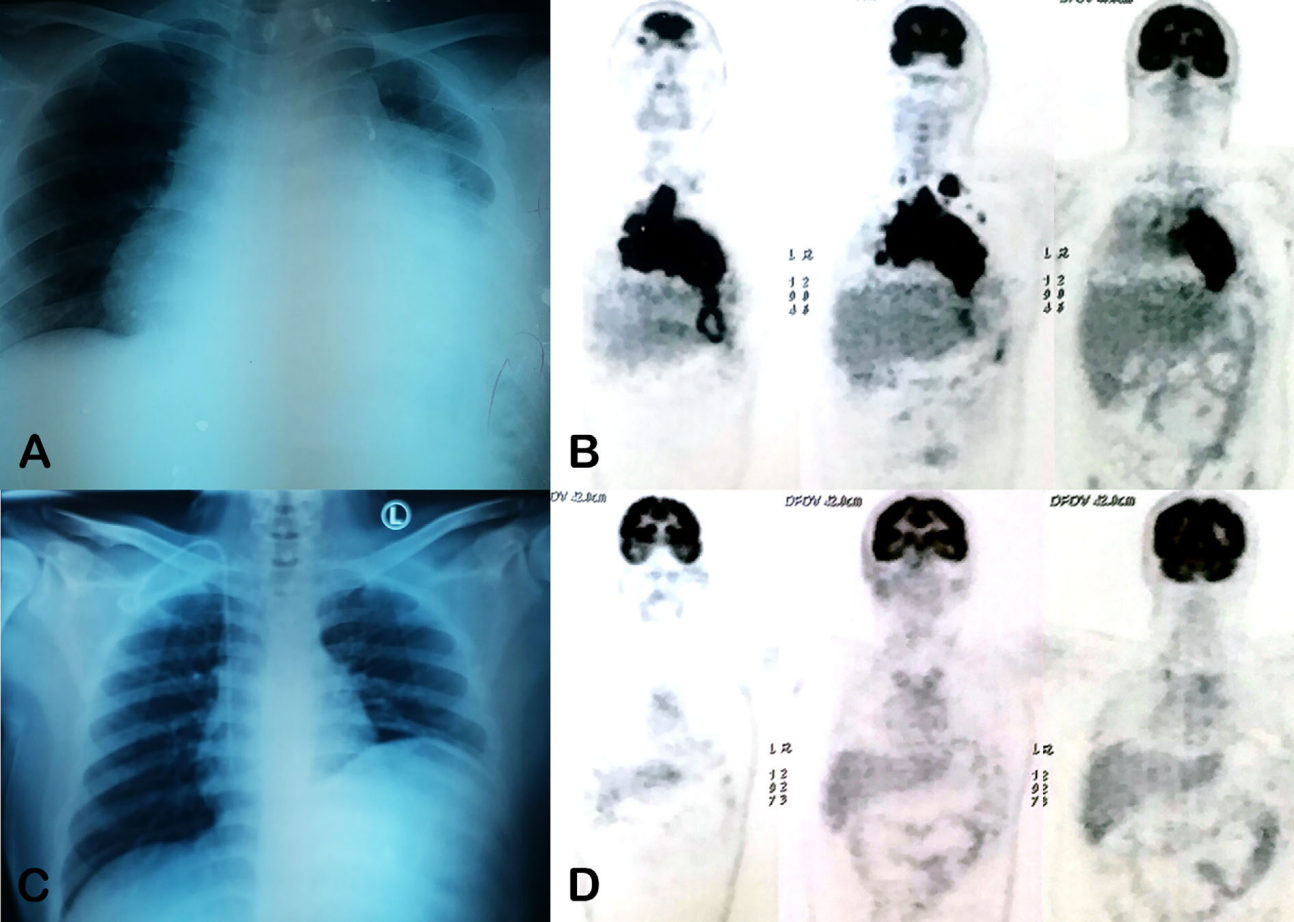
Comparison of tumour size before and after treatment by imaging. (A) Initial chest X‐ray. (B) Initial positron emission tomography (PET) scan image. (C) Post‐treatment chest X‐ray. (D) Post‐treatment PET scan.

We administered the Rituximab Dose‐Adjusted Etoposide Prednisolone Vincristine Cyclophosphamide Doxorubicin (R‐DA‐EPOCH) chemotherapy regimen every three weeks × six cycles. Even though the patient developed febrile neutropenia, nausea‐vomiting, alopecia, and peripheral neuropathy, the patient tolerated well to treatment and completely finished six cycles. After chemotherapy, improvement was recorded: clinical condition was improved and plain chest X‐ray radiography showed marked reduction in tumour size (Fig. 2). Furthermore, PET–CT imaging showed that the tumour size has reduced remarkably with significant decrease in metabolic activity. This patient was restaged to stage I and classified as partial response according to the International Working Group consensus response evaluation criteria in lymphoma (RECIL 2017). We proceeded to refer the patient for radiotherapy.

## Discussion

We reported a case of PMBCL in a young male patient, which in this case came to us with non‐specific symptoms such as shortness of breath, weight loss, and night sweat. The dyspnoeic appearance can be caused by combination of mass effect to adjacent structures (lung and vascular) and pleural or pericardial effusions [1]. Clinical manifestation may vary according to the primary site of the tumour [1]. Tumour in anterior or middle mediastinum may manifest as superior vena cava syndrome, dyspnoea, chest pain, or pleural/pericardial effusions. Mediastinal lymphoma in the posterior mediastinum may exert obstructive effects and even extends to the vertebral body producing a pathological fracture [1].

In cases of mediastinal lymphoma with multinodal involvement, it is very crucial to distinguish between primary mediastinal lymphomas with nodal extension and systemic lymphomas with mediastinal involvement. The incidence of primary mediastinal tumour is much less than secondary mediastinal involvement of systemic lymphoma [2]. Treatment strategies for PMBCL differ than those of systemic lymphomas. This type of malignant tumour is usually aggressive, fast growing, and highly infiltrative to adjacent structures [4].

In the initial imaging, the tumour had a largest diameter of 15.38 cm. Published reports find that in most patients, tumour diameter is >10 cm. Furthermore, leakage in the pericardial and pleural membrane is also prevalent and was observed in our patient. Core biopsy of the mediastinal mass provides excellent foundation for treatment as broad differential diagnosis could cause similar manifestation, such as thymoma, metastatic carcinomas, germ cell tumours, and other lymphomas with mediastinal nodes [4]. After histopathology confirms the diagnosis of non‐Hodgkin lymphoma, immunohistochemistry staining has utmost importance in determining the tumour's molecular subtype.

We utilized PET with fluorodeoxyglucose for pre‐treatment evaluation and response monitoring due to its high sensitivity in lymphoma [2]. The PET imaging evaluates not only the morphological decline of the tumour, but also the metabolic changes which often precede the size [2]. The maximum standardized uptake value (SUV) of the tumour in our patient was 20.6, which is far above the 3.4 cut‐off point for lymphoma, based on the data from a study conducted by Gawande and associates in a publication by Zuluaga, et al [2].

In general, patients with PMBCL shows favourable outcome and low rate of relapse [4]. In our case, the patient has low‐intermediate aaIPI, which could be translated to two‐year survival rate of 79% and relapse‐free survival of 74% as described by Shipp et al. [6]

There are several treatment regimens that has been used in clinical trials and daily practice. Combination of Rituximab with CHOP (R‐CHOP) chemotherapy is one of the common schedules in PMBCL with good prognosis features [5]. Published 2015 guidelines from the European Society of Medical Oncology (ESMO) suggest that R‐CHOP21 with radiotherapy should be effective in young low‐risk patients with bulky disease. However, clinical trials incorporating other regimens such as R‐DA‐EPOCH, R‐MACOP, MACOP‐B, R‐VACOP‐B, ProMACE‐CytaBOM, and NHL 15 also showed favourable results [3]. Current 2020 guidelines from the National Comprehensive Cancer Network (NCCN) suggest 6R‐DA‐EPOCH with radiotherapy for focal disease over R‐CHOP. We followed the guidelines and favourable response was observed. The residual mediastinal mass will undergo radiotherapy to achieve optimal response.

### Disclosure Statement

Appropriate written informed consent was obtained for publication of this case report and accompanying images.
